# Knowledge of University Students in Health Care Settings on Vaccines and Vaccinations Strategies: Impact Evaluation of a Specific Educational Training Course during the COVID-19 Pandemic Period in Italy

**DOI:** 10.3390/vaccines10071085

**Published:** 2022-07-06

**Authors:** Sara Boccalini, Alfredo Vannacci, Giada Crescioli, Niccolò Lombardi, Marco Del Riccio, Giuseppe Albora, Jonida Shtylla, Marco Masoni, Maria Renza Guelfi, Paolo Bonanni, Angela Bechini

**Affiliations:** 1 Department of Health Sciences, University of Florence, 50134 Florence, Italy; paolo.bonanni@unifi.it (P.B.); angela.bechini@unifi.it (A.B.); 2 Department of Neurosciences, Psychology, Drug Research and Child Health, Section of Pharmacology and Toxicology, University of Florence, 50134 Florence, Italy; alfredo.vannacci@unifi.it (A.V.); giada.crescioli@unifi.it (G.C.); niccolo.lombardi@unifi.it (N.L.); 3 Medical School of Hygiene and Preventive Medicine, University of Florence, 50134 Florence, Italy; marco.delriccio@unifi.it (M.D.R.); giuseppe.albora@unifi.it (G.A.); 4 SIAF—E-Learning Process Unit and IT Training, Area for the Innovation and Management of Information and Computer Systems, University of Florence, 50141 Florence, Italy; jonida.shtylla@unifi.it; 5 Department of Experimental and Clinical Medicine, University of Florence, 50134 Florence, Italy; marco.masoni@unifi.it (M.M.); mariarenza.guelfi@unifi.it (M.R.G.)

**Keywords:** medical education, elective training activities, immunization, impact assessment, health care workers

## Abstract

Background: Training future healthcare professionals on vaccination through specific courses is important to properly promote active immunization among the general population and to fight fake news and false beliefs on vaccinations. The aim of the study was to assess the impact of an elective course about vaccinations on the knowledge of medical students, pharmacy students, and medical resident in Hygiene and Preventive Medicine in Italy. Methods: The participants were asked to complete an anonymous questionnaire before and after an elective teaching activity (ETA) on vaccination. The two questionnaires contained the same 30 questions and focused on different aspects of vaccines and vaccination. The students who had attended the seminar were allowed to fulfil the post-lecture questionnaire. Both descriptive and inferential analysis were performed on the results; in particular, Student’s *t*-test for independent samples was used to compare the total score obtained before and after attending the ETA. Results: A total of 449 students participated in the ETA. Overall, the participation in the ETA allowed them to significantly improve their final score (+27.28%, *p* < 0.001). Good results were obtained even when comparing the three groups (medical students, pharmacy students and medical residents) separately. Females improved more than males, especially among pharmacy students. Discussion: The present study highlights the importance and the impact that extracurricular activities can have in improving knowledge about vaccinations. With vaccination and vaccine hesitancy and acceptance topics with increasing attention paid by the population, especially after the COVID-19 pandemic, it is fundamental to develop new strategies to increase future healthcare professionals’ knowledge about vaccinations.

## 1. Introduction

Vaccination has been defined as one of the greatest medical discoveries ever made and its impact on health is comparable to the access to drinking water for the population. In fact, these two preventive interventions have been very successful in decreasing infectious diseases and their related complications and deaths [1,2]. This success was reached thanks to high level of immunization coverage and its maintenance over time.

However, in Italy, as in other European countries, the phenomenon of “vaccine hesitancy” is growing. “Vaccine hesitancy” means the distrust that a part of the population has towards vaccines and vaccinations [3,4]. In a recent survey, about 15% of Italian parents of children aged 16–36 months resulted hesitant and less than 1% fully contrary to vaccines [5].

To contrast the vaccine hesitancy, populations need to receive correct and comprehensible information: the main source of information on vaccines usually are healthcare workers, as general practitioners, nurses, healthcare assistants and pharmacists [6]. Therefore, it is crucial to train university students—the future healthcare professionals—on vaccination through specific courses [7].

Several studies explored the knowledge of healthcare students on vaccination or the impact of specific educational interventions [8,9]. In Italy, attempts at increasing the level of knowledge among students in healthcare area have been recently carried on, and remarkable results were reported [10,11].

The main aim of the study was to assess the impact of a training experience on vaccines and vaccinations involving students enrolled in the single-cycle degrees in Medicine and Surgery, in Pharmacy, and in the postgraduate school of Hygiene and Preventive Medicine of the University of Florence (Italy) during the COVID-19 pandemic period, when the attention of the general population to prevention activities and new vaccines was at peak. In particular, in that period the first COVID-19 vaccines began to be available on the Italian market and to be administered to priority groups.

## 2. Material and Methods

An Elective Teaching Activity (ETA) on vaccines and vaccinations was organized at University of Florence (Italy) in the period February–April 2021. The lessons were held during the COVID-19 pandemic period through online platform Webex and students filled in the questionnaires through the learning management system (LMS) called “Moodle”. Moodle is an open-source platform, where it is possible to deposit and consult educational material, to process questionnaires and tasks, to support exercises, to follow lessons in video-streaming, and to use collaborative work tools.

The course (16 h) included lessons on the different types of vaccines, preclinical and clinical trials, regulatory process, process of vaccine production, supply, and storage, dispensation of vaccines, Health Technology Assessment (HTA) of new vaccines or vaccination strategies, national immunization plan, immunization coverage, impact of current immunization strategies, pharmaco-vigilance on vaccines and, lastly, fake news and scientific evidence on vaccines. A relevant focus was dedicated to development and authorization of COVID-19 vaccines, their characteristics and safety profile. Students from the III to VI year of the Degree Course in Medicine and Surgery and students from the III to V year of the Degree Course in Pharmacy in the academic year 2020–2021 decided to voluntarily attend these extracurricular lectures about vaccinations. Moreover, medical residents of the Medical School of Specialization in Hygiene and Preventive Medicine (I and II year) of the University of Florence attended the lessons too.

To evaluate the impact of the training intervention on the knowledge of the students on vaccinations, each participant was asked to fill in the same questionnaire before and after attending the ETA. Only those who attended the ETA were allowed to complete the post-lecture questionnaire. Both questionnaires contained 30 multi-choice questions on the main topics of the teaching activity. Each correct answer was assigned a score of 1.07 points, for a total score of 32 points that corresponded to “30/30 cum laude” (passing grade 18 points). The passing grade for each topic was reached with two correct answers out of the three questions contained in the topic.

Filling in the questionnaires was part of the educational evaluation activity of the course and ethical approval was not required. Moreover, collected data do not compromise students’ privacy since the questionnaires were focused on vaccine knowledge and no questions other than full name and university registration number were requested. At the end of the ETA students were asked to fill in a brief satisfaction questionnaire that was completely anonymous.

### Statistical Analysis

Results obtained before and after attending the ETA and results of the satisfaction questionnaire were analyzed. A descriptive statistical analysis was performed. Since variables were found to be normally distributed after have been tested through the Kolmogorov-Smirnov test, categorical data were reported as number and percentages and compared through the Chi-square test, while continuous data were reported as mean and standard deviation (SD), and compared with the Student’s *t*-test. Student’s *t*-test for paired data was performed in order to compare the total score obtained before and after attending the ETA. Results were considered statistically significant with a *p*-value of 0.05. All data were analyzed with STATA data analysis and statistical software version 17.0 (Copyright 1996–2022; Stata-Corp LP, College Station, TX, USA).

## 3. Results

Among a total number of about 1900 students eligible for this ETA (about 1450 medical students, about 420 pharmacy students, 23 trainees in Hygiene and Preventive Medicine), 449 students participated in the ETA on vaccines and vaccinations. Among them, 165 (36.8%) were students enrolled in the single-cycle degree in Pharmacy, 261 (58.1%) in the single-cycle degree in Medicine, and 23 (5.1%) in the postgraduate school of Hygiene and Preventive Medicine. Most students were females (*n* = 301, 67.0%), attending to the IV or V year of study (Table 1).

Overall, the participation in the ETA allowed students to improve their final score by 27.3% (*p* < 0.001; average improvement for individual students 26.59%). Pharmacy students reached a total pre-course score of 19/32 and a total post-course of 27/32 (+27.3%; *p* < 0.001), while Medicine students reached a total pre-course score of 22/32 and a total post-course of 31/32 (+27.6%; *p* < 0.001). Considering postgraduate students, their knowledge on vaccines and vaccinations improved by 10.8%, with a mean pre-course score of 26/32 and a mean post-course score of 30/32. T-student test for paired data showed statistically significant estimates in all student groups (Table 2).

Independently from the single-cycle degree/postgraduate course, both males and females improved their knowledge after attending the ETA. Females improved more than males, especially among Pharmacy students (Table 3).

Independently from the topic of each question, we observed a relatively low percentage of students who gave an incorrect answer during the post-course test (Table 4). The questions for which we observed a greater reduction in the total number of incorrect answers were: Question 1 (from 33.9% to 5.1%), Question 3 (from 35.6% to 5.1%), Question 7 (from 39.2% to 5.8%), Question 8 (from 47.2% to 15.0%), Question 9 (from 70.8% to 27.4%), Question 10 (from 54.8% to 12.2%), Question 12 (from 76.2% to 38.8%), Question 13 (from 34.5% to 2.7%), Question 14 (from 59.7% to 35.9%), Question 16 (from 59.2% to 27.4%), Question 20 (from 50.6% to 20.7%), and Question 23 (from 45.0% to 4.9%).

**Figure 1:**
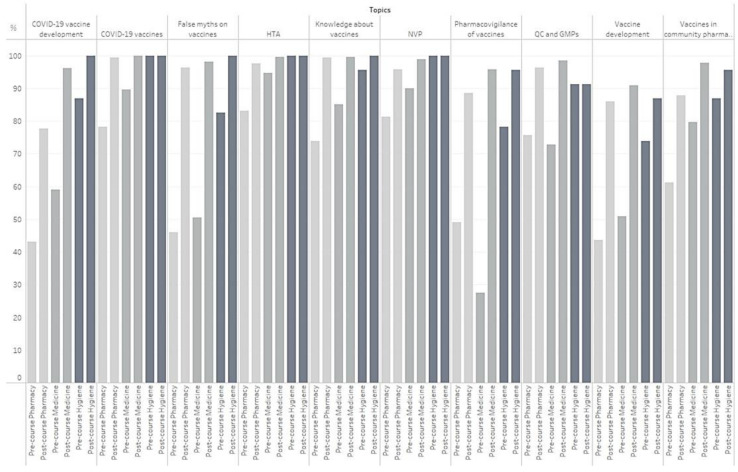
Percentages of students reaching the passing grade for each topic. GMP, good manufacturing practice; HTA, health technology assessment; NVP, national vaccination plan; QC, quality control.

Figure 1 shows the percentages of students reaching the passing grade for each topic. The two topics with a low percentage of students reaching the passing grade pre-ETA concerned “pharmacovigilance of vaccines” and “vaccines development”. Higher percentages of passing grade were observed post-ETA among postgraduate students (Hygiene).

Details of ETA topics, questions, answers, and differences between pre- and post-course test results among the three student groups are reported in Supplementary Tables S1–S10.

The ETA was much appreciated by the students, considering the responses they gave in the satisfaction questionnaire: in particular, 56.0% of them gave the maximum score when asked for an overall judgement, 4/4 on a Likert scale (the rest—44%—assigned 3 points out of 4). Moreover, most of them (53.0%) found the ETA “very useful” (4/4 on a Likert scale basis), while the rest (47.0%) found it “useful” (3/3). No negative answers (2/4, 1/4, 0/4) were registered.

## 4. Discussion

The presence and the importance of vaccinology in most Italian universities increased in recent years, after a long period in which this topic had insufficient dedicated time in university courses [7]. This growth has been made easier by the gradual choice of most Italian Universities to abandon the traditional educational system based on monographic courses and by the adoption of extracurricular activities (ETA). To graduate in Medicine and Surgery and in Pharmacy at the University of Florence, a student must attend a certain number of hours of ETAs that can be chosen among different activities (courses, seminars, laboratory projects, etc.) and topics, according to one’s own personal interests.

The aim of this study was to evaluate the impact of a vaccination-related ETA on students’ knowledge on this specific topic: this was assessed by administrating the students the same questionnaire before and after attending the course.

The results obtained suggest that the ETA was highly effective in increasing the students’ knowledge on vaccination: despite good overall scores in the pre-course test, the different groups were able to increase their final score considerably. Specifically, Pharmacy and Medicine students reported an increase of more than +27.0% between the pre- and the post-course tests (from 19.2/32 to 26.8/32 for pharmacy students, from 22.0/32 to 30.1/32 for Medicine students, *p* < 0.001). The improvement reported by the postgraduate students is also remarkable if considering the excellent knowledge of vaccine-related topics showed by the results of the pre-course test (their mean pre-course score was 26.3/32, while the mean post-course score was 29.6/32, *p* < 0.001). The success in strengthening vaccine learning was demonstrated by a remarkable increase in the percentage of post-course correct answers: no question—when considering the whole group of attendees—reported more wrong answers in the post-course questionnaire than in the pre-course questionnaire.

The traditional educational system (monographic course) does not appear to meet students’ expectations, and students often feel insufficiently prepared about the immunologic principles of vaccination and the epidemiology of vaccine-preventable diseases [12].

To enhance knowledge acquisition and to increase the attractivity towards vaccination or other topics, thematic summer camps—as those described by Vorsters et al. [13]—could be an interesting complement to the traditional academic curriculum.

Within the University context, extracurricular courses and activities such as the ETA presented in this manuscript become therefore particularly important, as they represent a powerful tool to increase medical students’ knowledge and engagement with the topic of vaccination. This should not be underestimated, as the importance of different sources of information on vaccines and vaccination has recently been highlighted due to its potential impact on the willingness to get vaccinated against COVID-19 or promote COVID-19 vaccination [14]. Moreover, ETAs are generally well accepted and welcomed by the students, as also shown by the results of the anonymous satisfactory questionnaire on the ETA presented in this manuscript.

The results of our study show indeed that training activities as the one that was held in April 2021 at the University of Florence could be considered an effective strategy to improve future healthcare workers’ knowledge about vaccinations. The importance of such interventions is dual: it is widely known that healthcare workers with better knowledge on vaccines and vaccination are more likely to get vaccinated than others [15]. This also applies to future healthcare workers, such as medical students, nursing students, and pharmacy students [16]. Moreover, a higher level of knowledge about vaccination is usually associated not only with a stronger willingness to be vaccinated but also with a higher chance to recommend appropriate vaccinations to patients [17,18].

This activity was offered and delivered during the COVID-19 pandemic and specifically in the very first period of the Italian COVID-19 vaccination campaign, which started in January 2021: in facts, the ETA was realized when the interest towards vaccinations was at its peak, when almost all healthcare workers have already been vaccinated and different groups (e.g., students in the healthcare area and teachers) in the population have started booking (or received) their first shot. This may somehow explain both the interest raised by this educational opportunity and the good knowledge shown by the students in the pre-test course.

This study presents certain limitations. As previously specified, the course was not mandatory but optional. This explains why the sample size is relatively small, if compared to the number of students attending the Medical School at the University of Florence. Moreover, for the same reason, it is possible that the success of this ETA was somehow driven by the strong personal interest shown by the participating students. Finally, it was not possible to enrich the study with more demographic data such as age, which would give a more in-depth understanding of the presented phenomenon.

## 5. Conclusions

This study deals with the attempt to organize, fulfil, and evaluate the impact of an ETA focused on vaccination which was directed to future pharmacists, medical doctors and specialists in Public Health and Preventive Medicine. We believe that a thorough knowledge on vaccines and vaccination will be increasingly required to future health care workers, especially after the COVID-19 pandemic and the rollout of COVID-19 vaccines, and therefore we consider these activities important to integrate knowledge and attitudes regarding vaccinations. The next generation of healthcare professionals should receive appropriate education, knowledge as well as technical skills on vaccines and vaccination, as this will help them recommend appropriate vaccinations to patients.

## Figures and Tables

**Table 1:** Students’ characteristics.

				Year of Study			
Faculty	Students N = 449 (%)	Males N = 148 (%)	Females N = 301 (%)	III	IV	V or More	Postgraduation
Pharmacy	165 (36.8)	38 (25.7)	127 (42.2)	15	44	106	-
Medicine	261 (58.1)	101 (68.2)	160 (53.1)	7	11	243	-
Hygiene	23 (5.1)	9 (6.1)	14 (4.7)	-	-	-	23

**Table 2:** Students’ scores in pre- and post-course tests.

Faculty	N Students (%)	Score Pre	Score Post	*p*-Value	Δ%
Pharmacy, mean ± SD	165 (36.8)	19.3 ± 4.3	26.9 ± 3.0	<0.001 *	+27.3%
Medicine, mean ± SD	261 (58.1)	22.0 ± 4.4	30.7 ± 2.9	<0.001 *	+27.6%
Hygiene, mean ± SD	23 (5.1)	26.3 ± 2.7	29.6 ± 3.4	<0.001 *	+10.8%
**Overall, mean ± SD**	**449**	**21.2 ± 4.6**	**29.2 ± 3.4**	<0.001 *	**+27.3%**

SD, standard deviation. * *t*-student test for paired data.

**Table 3:** Total scores pre- and post-ETA in males and females.

Faculty	N Students N = 449 (%)	Score Pre	*p*-Value	Score Post	*p*-Value	*p*-Value Pre-/Post-ETA
Pharmacy						
Males, mean ± SD	38 (25.7)	18.1 ± 4.3	0.052	25.9 ± 3.5	0.035	<0.001
Females, mean ± SD	127 (42.2)	19.6 ± 4.2		27.1 ± 2.8		<0.001
Medicine						
Males, mean ± SD	101 (68.2)	21.6 ± 4.7	0.191	30.7 ± 2.5	0.991	<0.001
Females, mean ± SD	160 (53.2)	22.3 ± 4.2		30.7 ± 3.1		<0.001
Hygiene						
Males, mean ± SD	9 (6.1)	26.6 ± 2.6	0.730	29.8 ± 2.7	0.854	0.012
Females, mean ± SD	14 (4.7)	26.1 ± 2.9		29.6 ± 2.1		0.005

ETA, elective teaching activity. *t*-Student test for paired data pre-/post-ETA.

**Table 4:** Incorrect answers given in pre- and post-course tests.

Incorrect Answers	Overall N = 449 (%)		Pharmacy N = 165 (%)		Medicine N = 261 (%)		Hygiene N = 23 (%)	
	Pre	Post	Pre	Post	Pre	Post	Pre	Post
**Question 1—Vaccines:**	**152 (33.9)**	**23 (5.1)**	**52 (31.5)**	**16 (9.7)**	**94 (36.0)**	**7 (2.7)**	**6 (26.1)**	**-**
Are comparable in all respects to drugs	93 (20.7)	15 (3.3)	29 (17.6)	10 (6.1)	58 (22.2)	5 (1.9)	6 (26.1)	-
Allow to treat people	8 (1.8)	-	1 (0.6)	-	7 (2.7)	-	-	-
All act only at the level of the individual vaccinated subject	51 (11.4)	8 (1.8)	22 (13.3)	6 (3.6)	29 (11.1)	2 (0.8)	-	-
**Question 2—Combined vaccines are:**	**48 (10.7)**	**5 (1.1)**	**25 (15.2)**	**3 (1.8)**	**22 (8.4)**	**2 (0.8)**	**1 (4.4)**	**-**
Produced using the recombinant DNA technique	25 (5.6)	3 (0.7)	13 (7.9)	3 (1.8)	11 (4.2)	-	1 (4.4)	-
Different vaccines administered in the same vaccination session but in different anatomical sites	10 (2.2)	1 (0.2)	3 (1.8)	-	7 (2.7)	1 (0.4)	-	-
Very effective but unfortunately they always result in an exponential increase in adverse events	13 (2.9)	1 (0.2)	9 (5.5)	-	4 (1.5)	1 (0.4)	-	-
**Question 3—Flu vaccines actually** **available in Italy are:**	**160 (35.6)**	**23 (5.1)**	**81 (49.1)**	**18 (10.9)**	**77 (29.5)**	**5 (1.9)**	**2 (8.7)**	**-**
Whole inactivated virus	127 (28.3)	18 (4.0)	67 (40.6)	14 (8.5)	58 (22.2)	4 (1.5)	2 (8.7)	-
Based on anatoxins	9 (2.0)	5 (1.1)	3 (1.8)	4 (2.4)	6 (2.3)	1 (0.4)	-	-
Based on polysaccharides	24 (5.4)	-	11 (6.7)	-	13 (5.0)	-	-	-
**Question 4—What was the first SARS-CoV-2 vaccine to be approved by EMA?**	**8 (1.8)**	**2 (0.4)**	**6 (3.6)**	**-**	**2 (0.8)**	**2 (0.8)**	**-**	**-**
Moderna	2 (0.5)	-	2 (1.2)	-	-	-	-	-
Oxford—AstraZeneca	4 (0.9)	1 (0.2)	4 (2.4)	-	-	1 (0.4)	-	-
Sanofi—GSK	1 (0.2)	-	-	-	1 (0.4)	-	-	-
J and J (Johnson and Johnson)	1 (0.2)	1 (0.2)	-	-	1 (0.4)	1 (0.4)	-	-
**Question 5—The Pfizer-Biontech vaccine differs from the Moderna vaccine in one of the following options:**	**127 (28.3)**	**21 (4.7)**	**66 (40.0)**	**14 (8.5)**	**60 (23.0)**	**7 (2.7)**	**1 (4.4)**	**-**
It does not need to be reconstituted with the physiological solution. unlike the Moderna vaccine which must be reconstituted	17 (3.8)	10 (2.2)	10 (6.1)	8 (4.9)	7 (2.9)	2 (0.8)	-	-
It exploits the mechanism of messenger RNA (mRNA). unlike the Moderna which is made up of purified antigens	60 (13.4)	2 (0.5)	35 (21.2)	2 (1.2)	24 (9.2)	-	1 (4.4)	-
It cannot be administered to subjects over 55 years of age	6 (1.3)	2 (0.5)	5 (3.0)	1 (0.6)	1 (0.4)	1 (0.4)	-	-
None of the above	44 (9.8)	7 (1.6)	16 (9.7)	3 (1.8)	28 (10.7)	4 (1.5)	-	-
**Question 6—How many doses of Pfizer-Biontech vaccine can be obtained from each vial. according to the latest AIFA legislation?**	**117 (26.1)**	**3 (0.7)**	**57 (34.6)**	**2 (1.2)**	**59 (22.6)**	**1 (0.4)**	**0 (4.4)**	**-**
1 dose	30 (6.7)	2 (0.5)	16 (9.7)	2 (1.2)	14 (5.4)	-	-	-
3 doses	24 (5.4)	-	14 (8.5)	-	10 (3.8)	-	-	-
4 doses	15 (3.3)	-	11 (6.7)	-	4 (1.5)	-	-	-
5 doses	48 (10.7)	1 (0.2)	16 (9.7)	-	31 (11.9)	1 (0.4)	1 (4.4)	-
**Question 7—Development phases of drugs and vaccines in which safety is also assessed are:**	**176 (39.2)**	**26 (5.8)**	**92 (55.8)**	**13 (7.9)**	**80 (30.6)**	**9 (3.4)**	**4 (17.4)**	**4 (17.4)**
Phases 1 and 2	62 (13.8)	10 (2.2)	20 (12.1)	3 (1.8)	39 (14.9)	4 (1.5)	3 (13.1)	3 (13.1)
Phases 2 and 3	59 (13.1)	10 (2.2)	29 (17.6)	6 (3.6)	29 (11.1)	3 (1.1)	1 (4.4)	1 (4.4)
Phase 4	55 (12.3)	6 (1.3)	43 (26.1)	4 (2.4)	12 (4.6)	2 (0.8)	-	-
**Question 8—The selection of adjuvants is performed:**	**212 (47.2)**	**67 (14.9)**	**77 (46.7)**	**30 (18.2)**	**126 (48.3)**	**28 (10.7)**	**9 (39.2)**	**9 (39.2)**
In phase 3	41 (9.1)	7 (1.6)	12 (7.3)	2 (1.2)	28 (10.7)	4 (1.5)	1 (4.4)	1 (4.4)
After vaccine authorisation	13 (2.9)	2 (0.5)	8 (4.9)	2 (1.2)	5 (1.9)	-	-	-
In phase 1-2	158 (35.2)	58 (12.9)	57 (34.5)	26 (15.8)	93 (35.6)	24 (9.2)	8 (34.8)	8 (34.8)
**Question 9—The first legislative rules on the development of vaccines date back to:**	**318 (70.8)**	**123 (27.4)**	**133 (80.6)**	**66 (40.0)**	**171 (65.5)**	**42 (16.1)**	**14 (60.9)**	**15 (9.0)**
1950	184 (41.0)	61 (13.6)	76 (46.1)	31 (18.8)	103 (39.5)	30 (11.5)	5 (21.7)	-
1970	97 (21.6)	16 (3.6)	46 (27.9)	9 (5.5)	47 (18.0)	6 (2.3)	4 (17.4)	1 (4.4)
1802	37 (8.2)	46 (10.2)	11 (6.7)	26 (15.8)	21 (8.1)	6 (2.3)	5 (21.7)	14 (60.9)
**Question 10—Which of these is one of the main ethical challenges in vaccination against SARS-CoV-2?**	**246 (54.8)**	**55 (12.2)**	**135 (76.8)**	**43 (26.1)**	**107 (41.0)**	**12 (4.6)**	**4 (17.4)**	**-**
Splitting of the doses	54 (12.0)	20 (4.5)	26 (15.8)	14 (8.5)	28 (10.7)	6 (2.3)	-	-
Adverse effects	62 (13.8)	16 (3.6)	31 (18.8)	15 (9.1)	31 (11.9)	1 (0.4)	-	-
mRNA technology	130 (29.0)	19 (4.2)	78 (42.3)	14 (8.5)	48 (18.4)	5 (1.9)	4 (17.4)	-
**Question 11—Which groups of subjects were excluded from pre-marketing testing of SARS-CoV-2 vaccines?**	**67 (14.9)**	**8 (1.8)**	**26 (15.8)**	**5 (3.0)**	**41 (15.7)**	**3 (1.1)**	**-**	**-**
Developing countries	43 (9.6)	6 (1.4)	13 (7.9)	4 (2.4)	30 (11.5)	2 (0.8)	-	-
Elderly people	10 (2.2)	-	5 (3.0)	-	5 (1.9)	-	-	-
Obese patients	14 (3.1)	2 (0.5)	8 (4.9)	1 (0.6)	6 (2.3)	1 (0.4)	-	-
**Question 12—Authorisation of the Comirnaty vaccine has been granted:**	**342 (76.2)**	**174 (38.8)**	**129 (78.2)**	**131 (79.4)**	**197 (75.5)**	**37 (14.1)**	**16 (69.6)**	**6 (26.1)**
Simultaneously on a global level	76 (16.9)	16 (3.6)	38 (23.0)	12 (7.3)	37 (14.2)	4 (1.5)	1 (4.4)	-
Firstly by the FDA in the USA	229 (51.0)	157 (35.0)	70 (42.4)	118 (71.5)	144 (55.2)	33 (12.5)	15 (65.2)	6 (26.1)
First. the Chinese government	37 (8.2)	1 (0.2)	21 (12.7)	1 (0.6)	16 (6.1)	-	-	-
**Question 13—How is the Quality Unit structured?**	**155 (34.5)**	**12 (2.7)**	**44 (26.6)**	**4 (2.4)**	**105 (40.2)**	**6 (2.3)**	**6 (26.1)**	**2 (8.7)**
Quality Control + Pharmacovigilance	139 (31.0)	12 (2.7)	36 (21.8)	4 (2.4)	97 (37.2)	6 (2.3)	6 (26.1)	2 (8.7)
Quality Assurance + Device monitoring	7 (1.6)	-	4 (2.4)	-	3 (1.1)	-	-	-
None of the above	9 (2.0)	-	4 (2.4)	-	5 (1.9)	-	-	-
**Question 14—GMP stands for:**	**268 (59.7)**	**161 (35.9)**	**116 (70.3)**	**108 (65.4)**	**141 (54.0)**	**44 (16.9)**	**11 (47.8)**	**9 (39.1)**
Standards of good manufacturing	12 (2.7)	4 (0.9)	9 (5.5)	4 (2.4)	3 (1.1)	-	-	-
Good Manufacturing Practices	251 (55.9)	156 (34.7)	103 (62.4)	104 (63.0)	137 (52.5)	43 (16.5)	11 (47.8)	9 (39.1)
Standards of good production practice	5 (1.1)	1 (0.2)	4 (2.4)	-	1 (0.4)	1 (0.4)	-	-
**Question 15—The materials for the production of a vaccine:**	**20 (4.5)**	**8 (1.8)**	**11 (6.7)**	**3 (1.8)**	**9 (3.4)**	**4 (1.5)**	**-**	**1 (4.4)**
Include only the raw materials purchased	4 (0.9)	-	1 (0.6)	-	3 (1.1)	-	-	-
Include only packaging materials	4 (0.9)	-	3 (1.8)	-	1 (0.4)	-	-	-
Are not analysed upon arrival and are stored at controlled temperature and humidity	12 (2.7)	8 (1.8)	7 (4.2)	3 (1.8)	5 (1.9)	4 (1.5)	-	1 (4.4)
**Question 16—In the pharmacy vaccines can be found:**	**266 (59.2)**	**123 (27.4)**	**111 (67.3)**	**92 (55.7)**	**145 (55.6)**	**26 (10.0)**	**10 (43.5)**	**5 (21.8)**
Only in the refrigerator	204 (45.4)	111 (24.7)	91 (55.2)	88 (53.3)	105 (40.2)	19 (7.3)	8 (34.8)	4 (17.4)
Only outside the refrigerator	1 (0.2)	-	1 (0.6)	-	-	-	-	-
All the above	61 (13.6)	12 (2.7)	19 (11.5)	4 (2.4)	40 (15.3)	7 (2.7)	2 (8.7)	1 (4.4)
**Question 17—The most frequent temperature range for thermolabile vaccines is:**	**108 (24.1)**	**9 (2.0)**	**48 (29.1)**	**2 (1.2)**	**59 (22.6)**	**7 (2.7)**	**1 (4.4)**	**-**
Below −15 °C	86 (19.2)	7 (1.6)	39 (23.6)	2 (1.2)	46 (17.6)	5 (1.9)	1 (4.4)	-
Between 15 °C and 25 °C	10 (2.2)	-	5 (3.0)	-	5 (1.9)	-	-	-
Between 8 °C and 15 °C	12 (2.7)	2 (0.5)	4 (2.4)	-	8 (3.1)	2 (0.8)	-	-
**Question 18—The cold chain includes:**	**92 (6.9)**	**35 (7.6)**	**55 (33.3)**	**29 (17.6)**	**34 (13.0)**	**5 (1.9)**	**3 (13.1)**	**-**
A final report of the load temperatures along the entire route and during storage in the pharmacy	20 (4.5)	6 (1.3)	11 (6.7)	3 (1.8)	7 (2.7)	3 (1.1)	2 (8.7)	-
Constant temperature monitoring by drivers and control centres	11 (2.5)	4 (0.9)	7 (4.2)	3 (1.8)	4 (1.5)	1 (0.4)	-	-
The use of temperature-controlled equipment	61 (13.6)	24 (5.4)	37 (22.4)	23 (13.9)	23 (8.8)	1 (0.4)	1 (4.4)	-
**Question 19—AEFI stands for:**	**116 (25.8)**	**8 (1.8)**	**47 (28.5)**	**2 (1.2)**	**64 (24.5)**	**6 (2.3)**	**5 (21.8)**	**-**
Association of Italian Exhibitions and Fairs	32 (7.1)	5 (1.1)	21 (12.7)	2 (1.2)	10 (3.8)	3 (1.1)	1 (4.4)	-
Adverse Events Following Injection	77 (17.2)	3 (0.7)	21 (12.7)	-	52 (19.9)	3 (1.1)	4 (17.4)	-
None of the above	7 (1.6)	-	5 (3.0)	-	2 (0.8)	-	-	-
**Question 20—Which of these features is NOT used to classify an AEFI:**	**227 (50.6)**	**93 (20.7)**	**102 (61.8)**	**73 (44.2)**	**118 (45.2)**	**18 (6.9)**	**7 (30.4)**	**2 (8.7)**
Errors in vaccination	182 (40.5)	48 (10.7)	64 (38.8)	36 (21.8)	111 (42.5)	11 (4.2)	7 (30.4)	1 (4.4)
Defects in the quality of the vaccine	23 (5.1)	10 (2.2)	18 (10.9)	5 (3.0)	5 (1.9)	5 (1.9)	-	-
Characteristics of the vaccine	22 (4.9)	35 (7.8)	20 (12.1)	32 (19.4)	2 (0.8)	2 (0.8)	-	1 (4.4)
**Question 21—To perform the causality assessment of an AEFI. the following is used:**	**91 (20.3)**	**43 (9.6)**	**82 (49.7)**	**25 (15.2)**	**108 (41.4)**	**16 (6.1)**	**8 (34.8)**	**2 (8.7)**
CIOMS/RUCAM algorithm	16 (3.6)	9 (2.0)	13 (7.9)	3 (1.8)	47 (18.0)	4 (1.5)	2 (8.7)	2 (8.7)
Schumock and Thornton algorithm	25 (5.6)	15 (3.3)	24 (14.6)	12 (7.3)	31 (11.9)	3 (1.1)	1 (4.4)	-
Naranjo scale	50 (11.1)	19 (4.2)	45 (27.3)	10 (6.1)	30 (11.5)	9 (3.4)	5 (21.7)	-
**Question 22—Which of the following statements is correct?**	**89 (19.8)**	**23 (5.1)**	**85 (51.5)**	**13 (7.9)**	**89 (34.1)**	**8 (3.1)**	**3 (13.1)**	**2 (8.7)**
Formaldehyde is used in vaccines as an adjuvant	55 (12.3)	15 (3.3)	53 (32.1)	10 (6.1)	47 (18.0)	5 (1.9)	1 (4.4)	-
The same amount of formaldehyde produced by an infant is present in vaccines	7 (1.6)	3 (0.7)	6 (3.6)	1 (0.6)	14 (5.4)	1 (0.4)	1 (4.4)	1 (4.4)
No vaccine contains formaldehyde	27 (6.0)	5 (1.1)	26 (15.8)	2 (1.2)	28 (10.7)	2 (0.8)	1 (4.4)	1 (4.4)
**Question 23—Which of the following statements is correct?**	**202 (45.0)**	**22 (4.9)**	**106 (64.2)**	**13 (10.6)**	**91 (15.7)**	**9 (3.4)**	**5 (21.8)**	**-**
At two months the child’s immune system is not already able to respond to vaccination	68 (15.1)	13 (2.9)	29 (17.6)	6 (3.6)	38 (14.6)	7 (2.7)	1 (4.4)	-
Vaccines weaken the immune system if administered too early	13 (2.9)	-	8 (4.9)	-	3 (1.1)	-	2 (8.7)	-
The newborn’s immune system is fragile and cannot be subjected to more than ten vaccinations in the first year of life	121 (27.0)	9 (2.0)	69 (41.8)	7 (4.2)	50 (19.2)	2 (0.8)	2 (8.7)	-
**Question 24—Which of the following statements is correct?**	**114 (25.4)**	**10 (2.2)**	**58 (35.1)**	**6 (3.6)**	**53 (20.3)**	**4 (1.5)**	**3 (13.1)**	**-**
Aluminium salts are used in vaccines as a preservative	85 (18.9)	9 (2.0)	39 (23.6)	6 (3.6)	43 (16.5)	3 (1.1)	3 (13.1)	-
The aluminum injected into the muscle with vaccines enters the blood immediately	7 (1.6)	-	4 (2.4)	-	3 (1.1)	-	-	-
Vaccines must not contain aluminium salts	22 (4.9)	1 (0.2)	15 (9.1)	-	7 (2.7)	1 (0.4)	-	-
**Question 25—According to the Italian National Immunization Plan 2017–2019. which of these vaccinations are recommended in pregnancy?**	**129 (28.7)**	**22 (4.9)**	**57 (34.6)**	**17 (10.3)**	**72 (27.6)**	**5 (1.9)**	**-**	**-**
Hepatitis B	29 (6.5)	-	11 (6.7)	-	18 (6.9)	-	-	-
Varicella (Chickenpox)	12 (2.7)	1 (0.2)	7 (4.2)	1 (0.6)	5 (1.9)	-	-	-
Measles-Mumps-Rubella	88 (19.6)	21 (4.7)	39 (23.6)	16 (9.7)	49 (18.8)	5 (1.9)	-	-
**Question 26—Which of the following vaccines are mandatory for school attendance under Law 119/2017 in Italy?**	**49 (10.9)**	**8 (1.8)**	**28 (17.0)**	**6 (3.6)**	**21 (8.0)**	**2 (0.8)**	**-**	**-**
Anti-meningococcal	39 (8.7)	3 (0.7)	20 (12.1)	2 (1.2)	19 (7.3)	1 (0.4)	-	-
Anti-influenza	6 (1.3)	4 (0.9)	6 (3.6)	4 (2.4)	-	-	-	-
Anti-pneumococcal	4 (0.9)	1 (0.2)	2 (1.2)	-	2 (0.8)	1 (0.4)	-	-
**Question 27—The impact of vaccination programmes is assessed through:**	**64 (14.3)**	**31 (6.9)**	**28 (17.0)**	**26 (15.8)**	**34 (13.0)**	**5 (1.9)**	**2 (8.7)**	**-**
Monitoring the hospitalizations trend	30 (6.7)	17 (3.8)	14 (8.5)	17 (10.3)	16 (6.1)	-	-	-
Monitoring of vaccination coverage	23 (5.1)	10 (2.2)	9 (5.5)	6 (3.6)	13 (5.0)	4 (1.5)	1 (4.4)	-
Monitoring the trend of mandatory disease notifications	11 (2.5)	4 (0.9)	5 (3.0)	3 (1.8)	5 (1.9)	1 (0.4)	1 (4.4)	-
**Question 28—A vaccine. to be included in the National Plan for Vaccine Prevention and. therefore. be offered actively and free of charge:**	**48 (10.7)**	**5 (1.1)**	**34 (20.6)**	**4 (2.4)**	**14 (5.4)**	**1 (0.4)**	**-**	**-**
It is sufficient that it is not too expensive	-	2 (0.5)	-	2 (1.2)	-	-	-	-
It is sufficient that has proven effective	18 (4.0)	1 (0.2)	17 (10.3)	1 (0.6)	1 (0.4)	-	-	-
It is sufficient that it has been shown to be safe	30 (6.7)	2 (0.5)	17 (10.3)	1 (0.6)	13 (5.0)	1 (0.4)	-	-
**Question 29—The HTA applied to vaccinations includes assessing:**	**72 (16.0)**	**3 (0.7)**	**41 (24.9)**	**2 (1.2)**	**31 (11.9)**	**1 (0.4)**	**-**	**-**
Organizational aspects	34 (7.6)	1 (0.2)	17 (10.3)	1 (0.6)	17 (6.5)	-	-	-
The ethical aspects	20 (4.5)	1 (0.2)	12 (7.3)	1 (0.6)	8 (3.1)	-	-	-
Possible alternative interventions	18 (4.0)	1 (0.2)	12 (7.3)	-	6 (2.3)	1 (0.4)	-	-
**Question 30—The economic evaluations of vaccinations show that:**	**59 (13.1)**	**19 (4.2)**	**42 (25.5)**	**15 (9.1)**	**17 (6.5)**	**3 (1.1)**	**-**	**1 (4.4)**
Vaccinations are only a cost to the NHS	15 (3.3)	5 (1.1)	10 (6.1)	3 (1.8)	5 (1.9)	1 (0.4)	-	1 (4.4)
There is no need to carry out economic assessments for vaccination	22 (4.9)	1 (0.2)	18 (10.9)	1 (0.6)	4 (1.5)	-	-	-
Only in some rare cases vaccination is cost-effective	22 (4.9)	13 (2.9)	14 (8.5)	11 (6.7)	8 (3.1)	2 (0.8)	-	-

AEFI, adverse event following immunization; HTA, health technology assessment; NHS, national health system.

## Data Availability

Data supporting reported results are available upon request to the corresponding author. Data were collected and managed in aggregated form according to European Union Regulation 2016/679 of European Parliament and the Italian Legislative Decree 2018/101.

## Supplementary Materials

The following supporting information can be downloaded at: https://www.mdpi.com/article/10.3390/vaccines10071085/s1, Table S1: Topic “Knowledge about vaccines”: test results pre- e post-course; Table S2: Topic “COVID-19 vaccines”: test results pre- e post-course; Table S3: Topic “Vaccine development”: test results pre- e post-course; Table S4: Topic “COVID-19 vaccine development”: test results pre- e post-course; Table S5: Topic “Quality Control (QC) and GMPs”: test results pre- e post-course; Table S6: Topic “Vaccines in community pharmacy”: test results pre- e post-course; Table S7: Topic “Pharmacovigilance of vaccines”: test results pre- e post-course; Table S8: Topic “False myths on vaccines”: test results pre- e post-course; Table S9: Topic “Italian national immunization plan (NIP)”: test results pre- e post-course; Table S10: Topic “HTA”: test results pre- e post-course.
