# Supplementary material for: Knowledge of University Students in Health Care Settings on Vaccines and Vaccinations Strategies: Impact Evaluation of a Specific Educational Training Course during the COVID-19 Pandemic Period in Italy
Source: Vaccines (Basel). 2022 Jul 6;10(7):1085. doi: 10.3390/vaccines10071085 (PMC9316295; doi:10.3390/vaccines10071085)
Supplement: Supplementary file 1 [file vaccines-10-01085-s001.zip › vaccines-1774550-supplementary.pdf]

Table S1. Topic "Knowledge about vaccines": test results pre- e post-course.

Questions n. 1-3	Answers	Pharmacy Pre-course N (%)	Pharmacy Post-course N (%)	Medicine Pre-course N (%)	Medicine Post-course N (%)	Hygiene Pre-course N (%)	Hygiene Post-course N (%)	*p-value* *	*p-value* **
Vaccines:<br>• *Are biological preparations*;<br>• Are comparable in all respects to drugs;<br>• Allow to treat people;<br>• All act only at the level of the individual vaccinated subject.	Incorrect	52 (31.71)	10 (6.10)	95 (36.40)	7 (2.68)	6 (26.09)	-	0.430	0.124
	Correct	112 (68.29)	154 (93.90)	166 (63.60)	254 (97.32)	17 (73.91)	23 (100)		
	Missing	1 (0.60)	1 (0.60)	-	-	-	-		
Combined vaccines are:<br>• Produced using the recombinant DNA technique;<br>• Different vaccines administered in the same vaccination session but in different anatomical sites;<br>• *Are constituted by the merging of several antigens or different vaccines in a single preparation;*<br>• Very effective but unfortunately they always result in an exponential increase in adverse events.	Incorrect	25 (15.24)	3 (1.82)	23 (8.81)	2 (0.77)	1 (4.35)	-	0.069	0.525
	Correct	139 (84.76)	162 (98.18)	238 (91.19)	259 (99.23)	22 (95.65)	23 (100)		
	Missing	1 (0.60)	-	-	-	-	-		
Flu vaccines actually available in Italy are:<br>• *Split virus. Sub-units and live attenuated virus*;<br>• Whole inactivated virus;<br>• Based on anatoxins;<br>• Based on polysaccharides.	Incorrect	81 (49.39)	18 (10.98)	80 (30.65)	5 (1.92)	2 (8.70)	-	0.000	0.000
	Correct	83 (50.61)	146 (89.02)	181 (69.35)	256 (98.08)	21 (91.30)	23 (100)		
	Missing	1 (0.60)	1 (0.60)	-	-	-	-		

Correct answers are in italics. * Difference in the distribution of correct/incorrect answers between Faculties pre-course. ** Difference in the distribution of correct/incorrect answers between Faculties post-course.

Table S2. Topic "COVID-19 vaccines": test results pre- e post-course.

Questions n. 4-6	Answers	Pharmacy Pre-course N (%)	Pharmacy Post-course N (%)	Medicine Pre-course N (%)	Medicine Post-course N (%)	Hygiene Pre-course N (%)	Hygiene Post-course N (%)	*p-value* *	*p-value* **
What was the first Sars-CoV-2 vaccine to be approved by EMA? • Oxford-Astrazeneca; • Moderna; • *Pfizer-Biontech*; • Sanofi-GSK; • J&J (Johnson & Johnson).	Incorrect	6 (3.66)	-	3 (1.15)	2 (0.77)	-	-	0.156	0.485
	Correct	158 (96.34)	165 (100)	258 (98.85)	259 (99.23)	23 (100)	23 (100)		
	Missing	1 (0.60)	-	-	-	-	-		
The Pfizer-Biontech vaccine differs from the Moderna vaccine in one of the following options: • It does not need to be reconstituted with the physiological solution. unlike the Moderna vaccine which must be reconstituted; • It exploits the mechanism of messenger RNA (mRNA). unlike the Moderna which is made up of purified antigens; • *An interval of at least 21 days between the first and the second dose is required. unlike Moderna vaccine which requires at least 28 days*; • It cannot be administered to subjects over 55 years of age; • None of the above.	Incorrect	66 (40)	13 (7.93)	64 (24.52)	8 (3.07)	1 (4.35)	-	0.000	0.038
	Correct	99 (60)	151 (92.07)	197 (75.48)	253 (96.03)	22 (95.65)	23 (100)		
	Missing	-	1 (0.60)	-	-	-	-		
How many doses of Pfizer-Biontech vaccine can be obtained from each vial. according to the latest AIFA legislation? • 1 dose; • 3 doses; • *6 doses;* • 5 doses; • 4 doses.	Incorrect	57 (34.76)	2 (1.21)	63 (24.14)	1 (0.38)	1 (4.35)	-	0.002	0.546
	Correct	107 (65.24)	163 (98.79)	198 (75.86)	260 (99.62)	22 (95.65)	23 (100)		
	Missing	1 (0.60)	-	-	-	-	-		

Correct answers are in italics. * Difference in the distribution of correct/incorrect answers between Faculties pre-course. ** Difference in the distribution of correct/incorrect answers between Faculties post-course.

Table S3. Topic "Vaccine development": test results pre- e post-course.

Questions n. 7-9	Answers	Pharmacy		Medicine		Hygiene		*p-value* *	*p-value* **
		**Pre-course N (%)**	**Post-course N (%)**	**Pre-course N (%)**	**Post-course N (%)**	**Pre-course N (%)**	**Post-course N (%)**		
Development phases of drugs and vaccines in which safety is also assessed are: • Phases 1 and 2; • Phases 2 and 3; • Phase 4; • *All of them.*	Incorrect	66 (40)	13 (7.88)	81 (31.03)	9 (3.45)	4 (17.39)	-	0.039	0.064
	Correct	99 (60)	152 (92.12)	180 (68.97)	252 (96.55)	19 (82.61)	23 (100)		
	Missing	-	-	-	-	-	-		
The selection of adjuvants is performed: • *In the preclinical phase;* • In phase 1-2; • In phase 3; • After vaccine authorisation.	Incorrect	77 (47.24)	30 (18.40)	133 (50.96)	28 (10.73)	9 (39.13)	3 (13.64)	0.473	0.082
	Correct	86 (52.76)	133 (81.60)	128 (49.04)	233 (89.27)	14 (60.87)	19 (86.36)		
	Missing	2 (1.21)	2 (1.21)	-	-	-	1 (4.35)		
The first legislative rules on the development of vaccines date back to: • 1950; • 1970; • *1902;* • 1802.	Incorrect	133 (81.60)	67 (41.10)	185 (70.88)	42 (16.09)	14 (63.64)	15 (68.18)	0.024	0.000
	Correct	30 (18.40)	96 (58.90)	76 (29.12)	219 (83.91)	8 (36.36)	7 (31.82)		
	Missing	2 (1.21)	2 (1.21)	-	-	1 (4.35)	1 (4.35)		

Correct answers are in italics. * Difference in the distribution of correct/incorrect answers between Faculties pre-course. ** Difference in the distribution of correct/incorrect answers between Faculties post-course.

Table S4. Topic "COVID-19 vaccine development": test results pre- e post-course.

**Questions n. 10-12**	**Answers**	**Pharmacy**		**Medicine**		**Hygiene**		*p-value* *	*p-value* **
		**Pre-course N (%)**	**Post-course N (%)**	**Pre-course N (%)**	**Post-course N (%)**	**Pre-course N (%)**	**Post-course N (%)**		
Which of these is one of the main ethical challenges in vaccination against SARS-Cov-2?<br>• Splitting of the doses;<br>• Adverse effects;<br>• mRNA technology;<br>• *Social inequalities.*	Incorrect	100 (61.73)	43 (26.38)	108 (41.38)	11 (4.21)	4 (17.39)	-	0.000	0.000
	Correct	62 (38.27)	120 (73.62)	153 (58.62)	250 (95.79)	19 (82.61)	23 (100)		
	Missing	3 (1.82)	2 (1.21)	-	-	-	-		
Which groups of subjects were excluded from pre-marketing testing of SARS-Cov-2 vaccines?<br>• *Pregnant and breastfeeding women;*<br>• Developing countries;<br>• Elderly people;<br>• Obese patients.	Incorrect	26 (15.85)	5 (3.05)	42 (16.19)	3 (1.15)	-	-	0.144	0.285
	Correct	138 (84.15)	159 (96.95)	219 (83.91)	258 (98.85)	23 (100)	23 (100)		
	Missing	1 (0.60)	1 (0.60)	-	-	-	-		
Authorisation of the Comirnaty vaccine has been granted:<br>• *Firstly from the MHRA in the UK;*<br>• Simultaneously on a global level;<br>• Firstly by the FDA in the USA;<br>• First. the Chinese government.	Incorrect	129 (80.63)	131 (79.88)	206 (78.93)	37 (14.18)	16 (69.57)	6 (26.09)	0.474	0.000
	Correct	31 (19.38)	33 (20.12)	55 (21.07)	224 (85.82)	7 (30.43)	17 (73.91)		
	Missing	5 (3.05)	1 (0.60)	-	-	-	-		

Correct answers are in italics. * Difference in the distribution of correct/incorrect answers between Faculties pre-course. ** Difference in the distribution of correct/incorrect answers between Faculties post-course.

Table S5. Topic "Quality Control (QC) and GMPs": test results pre- e post-course.

Questions n. 13-15	Answers	Pharmacy Pre-course N (%)	Pharmacy Post-course N (%)	Medicine Pre-course N (%)	Medicine Post-course N (%)	Hygiene Pre-course N (%)	Hygiene Post-course N (%)	*p-value* *	*p-value* **
How is the Quality Unit structured?<br>• *Quality Control + Quality Assurance;*<br>• Quality Control + Pharmacovigilance;<br>• Quality Assurance + Device Monitoring;<br>• None of the above.	Incorrect	44 (26.83)	4 (2.42)	120 (45.98)	6 (2.30)	6 (26.09)	2 (8.70)	0.000	0.184
	Correct	120 (73.17)	161 (97.58)	141 (54.02)	255 (97.70)	17 (73.91)	21 (91.30)		
	Missing	1 (0.60)	-	-	-	-	-		
GMP stands for:<br>• Standards of good manufacturing;<br>• Good Manufacturing Practices;<br>• Standards of good production practice;<br>• *All the above.*	Incorrect	116 (70.30)	108 (65.45)	147 (56.32)	44 (16.86)	11 (47.83)	9 (39.13)	0.006	0.000
	Correct	49 (29.70)	57 (43.55)	114 (43.68)	217 (83.14)	12 (52.17)	14 (60.87)		
	Missing	-	-	-	-	-	-		
The materials for the production of a vaccine:<br>• Include only the raw materials purchased;<br>• *Are analysed upon arrival and stored at controlled temperature and humidity;*<br>• Include only packaging materials;<br>• Are not analysed upon arrival and are stored at controlled temperature and humidity.	Incorrect	11 (6.79)	3 (1.83)	15 (5.75)	4 (1.53)	-	1 (4.35)	0.428	0.619
	Correct	151 (93.21)	161 (98.17)	246 (94.25)	257 (98.47)	23 (100)	22 (95.65)		
	Missing	3 (1.83)	1 (0.60)	-	-	-	-		

Correct answers are in italics. * Difference in the distribution of correct/incorrect answers between Faculties pre-course. ** Difference in the distribution of correct/incorrect answers between Faculties post-course.

Table S6. Topic "Vaccines in community pharmacy": test results pre- e post-course.

Questions n. 16-18	Answers	Pharmacy Pre-course N (%)	Pharmacy Post-course N (%)	Medicine Pre-course N (%)	Medicine Post-course N (%)	Hygiene Pre-course N (%)	Hygiene Post-course N (%)	*p-value* *	*p-value* **
In the pharmacy vaccines can be found: • Only in the refrigerator; • Only outside the refrigerator; • *Either in or out of the refrigerator;* • All the above.	Incorrect	111 (67.68)	93 (56.36)	148 (56.70)	26 (9.96)	10 (42.48)	5 (21.74)	0.020	0.000
	Correct	53 (32.32)	72 (43.64)	113 (43.30)	235 (90.04)	13 (56.52)	18 (78.26)		
	Missing	1 (0.60)	-	-	-	-	-		
The most frequent temperature range for thermolabile vaccines is: • Below -15°C; • *Between 2°C and 8°C;* • Between 8°C and 15°C; • Between 15°C and 25°C.	Incorrect	48 (29.45)	2 (1.21)	63 (24.14)	7 (2.68)	1 (4.35)	-	0.030	0.453
	Correct	115 (70.55)	163 (98.79)	198 (75.86)	254 (97.32)	22 (95.65)	22 (100)		
	Missing	2 (1.21)	-	-	-	-	1 (4.35)		
The cold chain includes: • The use of temperature-controlled equipment; • The transport of vaccines cannot be carried out with normal means of transport; • Constant temperature monitoring by drivers and control centres; • A final report of the load temperatures along the entire route and during storage in the pharmacy; • *All the above.*	Incorrect	55 (33.54)	29 (17.68)	38 (14.56)	4 (1.53)	3 (13.04)	-	0.000	0.000
	Correct	109 (66.46)	135 (82.32)	223 (85.44)	257 (98.47)	20 (86.96)	23 (100)		
	Missing	1 (0.60)	1 (0.60)	-	-	-	-		

Correct answers are in italics. * Difference in the distribution of correct/incorrect answers between Faculties pre-course. ** Difference in the distribution of correct/incorrect answers between Faculties post-course.

Table S7. Topic "Pharmacovigilance of vaccines": test results pre- e post-course.

Questions n. 19-21	Answers	Pharmacy		Medicine		Hygiene		*p-value* *	*p-value* **
		**Pre-course N (%)**	**Post-course N (%)**	**Pre-course N (%)**	**Post-course N (%)**	**Pre-course N (%)**	**Post-course N (%)**		
AEFI stands for:<br>• Association of Italian Exhibitions and Fairs [Associazione Esposizioni e Fiere Italiane];<br>• Adverse Events Following Injection;<br>• *Adverse Events Following Immunisation*;<br>• None of the above.	Incorrect	47 (28.83)	2 (1.21)	67 (25.67)	6 (2.30)	5 (21.74)	-	0.667	0.571
	Correct	116 (71.17)	163 (98.79)	194 (74.33)	255 (97.70)	18 (78.26)	23 (100)		
	Missing	2 (1.21)	-	-	-	-	-		
Which of these features is NOT used to classify an AEFI:<br>• *Errors in vaccination*;<br>• Age of the patient;<br>• Defects in the quality of the vaccine;<br>• Characteristics of the vaccine.	Incorrect	102 (61.82)	73 (44.51)	170 (65.13)	18 (6.90)	7 (30.43)	2 (8.70)	0.004	0.000
	Correct	63 (38.18)	91 (55.49)	91 (34.87)	243 (93.10)	16 (69.57)	21 (91.30)		
	Missing	-	1 (0.60)	-	-	-	-		
To perform the causality assessment of an AEFI. The following is used:<br>• CIOMS/RUCAM algorithm;<br>• Schumock & Thornton algorithm;<br>• Naranjo scale;<br>• *WHO algorithm*.	Incorrect	82 (52.90)	25 (15.24)	108 (41.38)	16 (6.13)	8 (34.78)	2 (8.70)	0.165	0.008
	Correct	73 (47.10)	139 (84.76)	131 (50.19)	245 (93.87)	15 (65.22)	21 (91.30)		
	Missing	10 (6.06)	1 (0.60)	22 (8.43)	-	-	-		

Correct answers are in italics. * Difference in the distribution of correct/incorrect answers between Faculties pre-course. ** Difference in the distribution of correct/incorrect answers between Faculties post-course.

Table S8. Topic "False myths on vaccines": test results pre- e post-course.

Questions n. 22-24	Answers	Pharmacy		Medicine		Hygiene		*p-value* *	*p-value* **
		Pre-course N (%)	Post-course N (%)	Pre-course N (%)	Post-course N (%)	Pre-course N (%)	Post-course N (%)		
Which of the following statements is correct?<br>• *Formaldehyde is contained in a small number of vaccines but in minimal quantities (<0.1 mg)*;<br>• Formaldehyde is used in vaccines as an adjuvant;<br>• The same amount of formaldehyde produced by an infant is present in vaccines;<br>• No vaccine contains formaldehyde.	Incorrect	85 (52.15)	13 (7.98)	89 (34.10)	9 (3.45)	3 (13.04)	2 (8.70)	0.001	0.101
	Correct	78 (47.85)	150 (92.02)	168 (64.37)	252 (96.55)	20 (86.06)	21 (91.30)		
	Missing	2 (1.21)	2 (1.21)	4 (1.53)	-	-	-		
Which of the following statements is correct?<br>• At two months the child's immune system is not already able to respond to vaccination;<br>• Vaccines weaken the immune system if administered too early;<br>• The newborn's immune system is fragile and cannot be subjected to more than ten vaccinations in the first year of life;<br>• *The newborn's immune system has great potential: it could respond to over 10.000 vaccines at the same time.*	Incorrect	106 (65.84)	13 (7.93)	99 (37.93)	10 (3.83)	5 (21.74)	-	0.000	0.092
	Correct	55 (34.16)	151 (92.07)	162 (62.07)	251 (96.17)	18 (78.26)	23 (100)		
	Missing	4 (2.45)	1 (0.60)	-	-	-	-		
Which of the following statements is correct?<br>• *Aluminium salts are used in vaccines to enhance immune response*;<br>• Aluminium salts are used in vaccines as a preservative;<br>• The aluminium injected into the muscle with vaccines enters the blood immediately;<br>• Vaccines must not contain aluminium salts.	Incorrect	58 (35.80)	6 (3.64)	55 (21.07)	4 (1.53)	3 (13.04)	-	0.001	0.272
	Correct	104 (64.20)	159 (96.36)	206 (78.93)	257 (98.47)	20 (86.96)	23 (100)		
	Missing	3 (1.82)	-	-	-	-	-		

Correct answers are in italics. * Difference in the distribution of correct/incorrect answers between Faculties pre-course. ** Difference in the distribution of correct/incorrect answers between Faculties post-course.

Table S9. Topic "Italian national immunization plan (NIP)": test results pre- e post-course.

Questions n. 25-27	Answers	Pharmacy Pre-course N (%)	Pharmacy Post-course N (%)	Medicine Pre-course N (%)	Medicine Post-course N (%)	Hygiene Pre-course N (%)	Hygiene Post-course N (%)	*p-value* *	*p-value* **
According to the Italian National Immunization Plan 2017-19. which of these vaccinations are recommended in pregnancy? • *Influenza and diphtheria-tetanus-pertussis;* • Hepatitis B; • Varicella (Chickenpox); • Measles-Mumps-Rubella.	Incorrect	57 (34.55)	17 (10.30)	74 (28.35)	5 (1.92)	-	-	0.003	0.000
	Correct	108 (65.45)	148 (89.70)	187 (71.65)	256 (98.08)	23 (100)	23 (100)		
	Missing	-	-	-	-	-	-		
Which of the following vaccines are mandatory for school attendance under Law 119/2017 in Italy? • Anti-pneumococcal; • Anti-influenza; • *Hexavalent and MPRV;* • Anti-meningococcal.	Incorrect	28 (16.97)	6 (3.66)	22 (8.43)	2 (0.77)	-	-	0.005	0.073
	Correct	137 (83.03)	158 (96.34)	239 (91.57)	259 (99.23)	23 (100)	23 (100)		
	Missing	-	1 (0.60)	-	-	-	-		
The impact of vaccination programmes is assessed through: • Monitoring the trend of mandatory disease notifications; • Monitoring of vaccination coverage; • *The monitoring of the disease burden (notifications and hospitalizations) based on the vaccination coverage achieved;* • Monitoring the hospitalizations trend.	Incorrect	28 (17.18)	26 (15.76)	37 (14.18)	5 (1.92)	2 (8.70)	-	0.481	0.000
	Correct	135 (82.82)	139 (84.24)	224 (85.82)	256 (98.08)	21 (91.30)	23 (100)		
	Missing	2 (1.21)	-	-	-	-	-		

Correct answers are in italics. * Difference in the distribution of correct/incorrect answers between Faculties pre-course. ** Difference in the distribution of correct/incorrect answers between Faculties post-course.

Table S10. Topic "HTA": test results pre- e post-course.

Questions n. 28-30	Answers	Pharmacy Pre-course N (%)	Pharmacy Post-course N (%)	Medicine Pre-course N (%)	Medicine Post-course N (%)	Hygiene Pre-course N (%)	Hygiene Post-course N (%)	*p-value* *	*p-value* **
A vaccine. To be included in the National Plan for Vaccine Prevention and. Therefore. Be offered actively and free of charge: • *Must be assessed according to the HTA approach;* • It is sufficient that it is not too expensive; • It is sufficient that has proven effective; • It is sufficient that it has been shown to be safe.	Incorrect	34 (20.61)	4 (2.45)	20 (7.66)	1 (0.38)	-	-	0.000	0.125
	Correct	131 (79.39)	159 (97.55)	241 (92.34)	260 (99.62)	23 (100)	23 (100)		
	Missing	-	2 (1.21)	-	-	-	-		
The HTA applied to vaccinations includes assessing: • Organisational aspects; • The ethical aspects; • Possible alternative interventions; • *All the above.*	Incorrect	41 (25.15)	2 (1.21)	36 (13.79)	1 (0.38)	-	-	0.001	0.539
	Correct	122 (74.85)	161 (98.77)	255 (86.21)	(99.62)	23 (100)	23 (100)		
	Missing	2 (1.21)	2 (1.21)	-	-	-	-		
The economic evaluations of vaccinations show that: • There is no need to carry out economic assessments for vaccination; • Only in some rare cases vaccination is cost-effective; • *Vaccinations are an investment for the NHS (National Health System);* • Vaccinations are only a cost to the NHS.	Incorrect	42 (25.77)	11 (6.75)	21 (8.05)	2 (0.71)	-	1 (4.35)	0.000	0.003
	Correct	121 (74.23)	152 (93.25)	240 (91.95)	259 (99.23)	23 (100)	22 (95.65)		
	Missing	2 (1.21)	2 (1.21)	-	-	-	-		

Correct answers are in italics. * Difference in the distribution of correct/incorrect answers between Faculties pre-course. ** Difference in the distribution of correct/incorrect answers between Faculties post-course.
